# Leveraging Transcriptome Insights and *GsHZ4* Gene Expression to Improve Alkaline Tolerance in *Lupinus angustifolius*

**DOI:** 10.3390/plants14223408

**Published:** 2025-11-07

**Authors:** Jixiang Tang, Mengyu Liu, Yujing Liu, Xiaoyu Wang, Xinlei Du, Xinyao Liu, Mengyue Zhou, Honglin Mao, Yixuan Feng, Qing Gao, Junfeng Zhang, Lei Cao

**Affiliations:** 1 College of Horticulture and Landscape Architecture, Northeast Agricultural University, No. 600, Changjiang Road, Xiangfang District, Harbin 150030, China; jxtang1019@163.com (J.T.); liumm0730@163.com (M.L.); yujingliu0000@163.com (Y.L.); wxy766696114@163.com (X.W.); duxinlei0101@163.com (X.D.); liuxinyao000927@163.com (X.L.); 15995318092@163.com (M.Z.); 18345749688@163.com (H.M.); 18846043687@163.com (Y.F.); 13039763390@163.com (Q.G.); jfzhang1989@163.com (J.Z.); 2 School of Geography and Tourism, Harbin University, No. 109, Zhongxing Avenue, Nangang District, Harbin 150076, China

**Keywords:** *Lupinus angustifolius*, *Glycine soja*, RNA-seq, *GsHZ4*, alkaline stress

## Abstract

Soil alkalinity severely restricts the cultivation of *Lupinus angustifolius*, a valuable legume. Wild soybean (*Glycine soja*) is a leguminous plant with extremely strong alkaline resistance (pH 8.5). Transferring the alkali-tolerant genes from wild soybeans into lupinus can effectively enhance the alkali tolerance. In this study, we combined transcriptome profiling and genetic transformation to elucidate the molecular basis of alkaline stress response in lupinus. RNA-seq analysis of root tips under acid (HCl, pH 4.0) and alkali (NaHCO_3_, pH 8.5) stress revealed 104,353 annotated unigenes, with differential expression patterns highlighting enrichment in cellular component, binding, and catalytic activity categories. KEGG pathway analysis indicated that early responses involved ribosome-related pathways, while later stages activated plant hormone signaling and MAPK pathways. Notably, no homeodomain-leucine zipper (HD-Zip) family genes were identified in the lupinus genome. Therefore, we transferred GsHZ4, an alkali-resistant HD-Zip transcription factor from wild soybean into lupinus hairy roots via *Agrobacterium rhizogenes*-mediated transformation. Overexpression of *GsHZ4* significantly enhanced antioxidant enzyme activities (CAT, POD, and SOD) and reduced malondialdehyde content under NaHCO_3_ stress. Furthermore, the promoter of *GsHZ4* expression was strongly induced by indole-3-acetic acid (IAA). Key alkali-responsive genes (*LaKIN*, *LaMYB34*, *LaDnaJ1*, *LaDnaJ20*, *LaNAC22*, and *LaNAC35*) were upregulated in transgenic lines, suggesting that *GsHZ4* integrates into the endogenous stress-regulation network. Our findings demonstrate that heterologous expression of *GsHZ4* can enhance alkaline tolerance of lupinus, providing a novel strategy for breeding stress-resistant varieties and expanding lupinus cultivation in saline–alkali soils.

## 1. Introduction

Saline–alkali soils restrict agricultural production (crop growth and yields) and development in China, an issue that has been a focus of research [1]. Relative to salt stress, alkali stress involves higher level pH; it usually causes Na^+^, HCO_3_^−^, SO_3_^2−^, and CO_3_^2−^ toxicity. However, the mechanism of plant alkali tolerance is not fully understood [2]. Lupinus (*Lupinus angustifolius)*, a member of the butterfly flower subfamily of the legume family, has palmate compound leaves with 5–9 leaflets and 5–10 cm-long terminal racemes. This colorful plant is used widely in landscaping. Lupinus leaves are rich in protein and are good source of essential amino acids for animals. They are used widely in chicken, duck, cow, rabbit, and pig feed to improve meat and milk production quality [3]. Lupinus roots could use solid phosphorus from the soil and fix nitrogen through rhizobia, improving soil nutrition. Thus, lupinus has high economic value. Compared to neutral or alkaline soil, lupinus is more suitable for growing in slightly acidic soil [4]. Adapting/Adjusting soil pH during planting is difficult, it often requires soil amendment by applying sulfur powder, which complicates the process and increases the costs of introduction and cultivation. This study was conducted to examine lupinus’ alkaline resistance, with the aim of increasing the lupinus planting area and improving the utilization of saline–alkali soil.

The publication of the lupinus genome has facilitated molecular breeding efforts aimed at increasing the plant’s alkaline resistance and reducing its reliance on acidic soil. In 2019, the lupinus genome was reported [5], enabling the small-scale adaptation of the species [6]. Lupinus transformation via *Agrobacterium rhizogenes* has been described [7]. Alkali stress inhibits lupinus growth and nutrient absorption, and the strong buffering capacity of HCO_3_^−^ is a key factor determining the survival of lupinus in calcareous soil [4]. The treatment of farmland soil with filter mud improved the growth, seed alkaloid content, and yield of lupinus termis by upregulating the plant-water relationship [8]. Previous research had focused on the molecular mechanism of solid phosphorus utilization by lupinus.

Wild soybean (*Glycine soja*) has strong resistance to stress and can grow under extreme conditions [9]. Its genes have been used to improve the salt–alkali resistance of cultivated soybean [10,11]. Lupinus and wild soybean are leguminous plants. Thus, the transfer of wild soybean genes related to adaptation to specific environments into related flowering legumes and forage is an efficient and reliable means of improving these varieties. Exogenous genes can improve the abiotic stress tolerance of model plants. *GmWRKY16* enhances the abiotic tolerance of *Arabidopsis* [12]. The overexpression of *GmUBC2* enhances the drought tolerance of *Arabidopsis* [13]. The overexpression of the rice gene *SNAC1* improves the salt tolerance of transgenic cotton [14]. *ZmmiR156* improves the drought and salt resistance of tobacco [15]. Wild soybean genes can be transferred into lupinus to improve its alkaline stress tolerance. In this study, we transferred wild soybean genes into lupinus hairy roots, where they were expressed normally. These exogenous genes responded to stress and hormones by regulating peroxide metabolism and endogenous stress-tolerance genes in lupinus.

The HB family constitutes a superfamily of transcription factors characterized by a conserved homeodomain, playing pivotal roles in plant embryonic development, organogenesis, and cell differentiation [16]. Its subfamily, HD-Zip, defined by the distinctive homeodomain-leucine zipper (HD-LZ) motif, functions through dimerization to bind DNA. It is further categorized into four classes (I–IV), which specifically regulate diverse processes including stress responses, photomorphogenesis, vascular development, and epidermal differentiation, representing a unique and crucial group of plant-specific regulatory factors. The HB genes share a homologous protein domain with a conserved 60-amino-acid motif that always interacts specifically with DNA sequences from downstream genes’ promoters. Members of the HD-Zip family have a leucine zipper (LZ) motif immediately downstream of the HD. The HD is responsible for specific binding to DNA, and the LZ acts as a dimerization motif. HD-Zip proteins bind to DNA as dimers, and the absence of an LZ abolishes their binding ability. HD-Zip genes participate in plant responses to salt stress by activating the antioxidant system, regulating osmotic homeostasis, and maintaining Na^+^/K^+^ homeostasis. For example, the HD-ZIP I gene *MdHB7*, found in apples, reduces the H_2_O_2_ and O_2_^−^ concentrations and increases superoxide dismutase (SOD) and peroxidase (POD) activity, thereby reducing oxidative damage under salt stress [17]. The heterologous overexpression of the pepper gene *CaHB1* in tomato improves photosynthesis and stress resistance [18]. Lupinus was found to have some genes homologous to those of wild soybean, but to lack alkali tolerance genes such as *GsHZ4*. This genomic difference may be attributable to evolution and may underlie the difference in tolerance. The *GsHZ4* gene was derived from wild soybean strain *G07256*, which has strong saline–alkali resistance. Expression pattern and tissue and subcellular localization analyses and yeast one-hybrid assays indicated that *GsHZ4* is an HD-Zip transcription factor that binds to the *GsNAC019* promoter and improves plants’ alkali resistance [9,19]. It improved this resistance in lupinus, and this approach represents a new strategy enabling the expansion of lupinus cultivation in saline–alkali soil.

## 2. Results

### 2.1. Lupinus Responses to Acid and Alkali Treatments

The DNBSEQ platform was used to measure a total of 95.77 Gb of data. After assembly and redundancy removal, 104,353 unigenes were obtained, with a total length, average length, N50, and GC content of 166,491,728 bp, 1595 bp, 2183 bp, and 40.45%, respectively (Table 1). Quality statistics for the filtered reads and the results of reference genome alignment are provided in Table 2 and Table 3, respectively. The comparison of the unigenes with seven functional annotation databases yielded values of 94,646 (90.70%) for NR, 92,967 (89.09%) for NT, 75,069 (71.94%) for SwissProt, 77,746 (74.50%) for KOG, 76,181 (73.00%) for KEGG, 77,940 (74.69%) for GO, and 76,334 (73.15%) for Pfam (Table 4). In total, 81,056 CDSs were detected using Transdecoder and 4618 unigenes encoding transcription factors were predicted.

**Figure 1:**
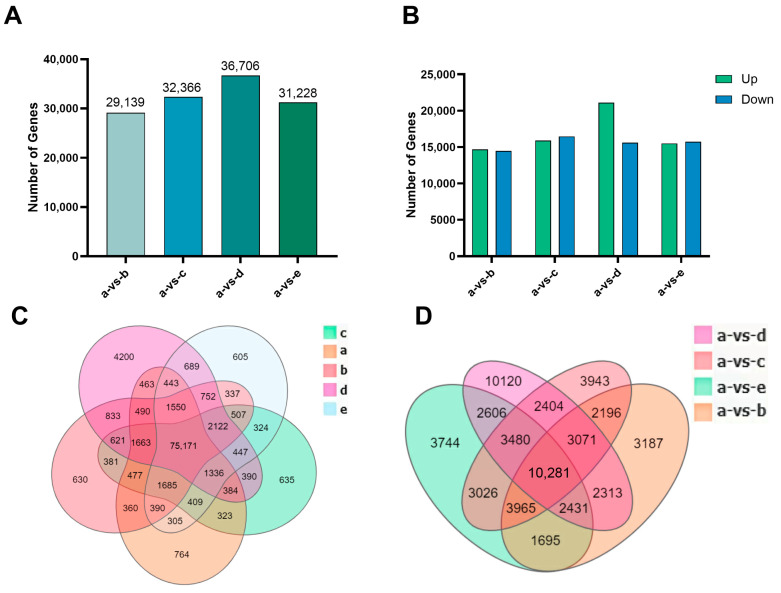
Differential expressed genes analysis after transcriptome sequencing. (**A**) The number of DEGs in each group compared with the control group; (**B**) the number of upregulated (green columns) and down-regulated (blue columns) genes in all DEGs in each group compared with the control group. (**C**) Venn map of DEGs numbers in each group. (**D**) Venn map of DEG numbers in each group compared with the control group. Group ‘a’ represents the control group, ‘b’ represents the acid treatment for 3 h, ‘c’ represents the acid treatment for 6 h, ‘d’ represents the alkali treatment for 3 h, and ‘e’ represents the alkali treatment for 6 h.

**Figure 2:**
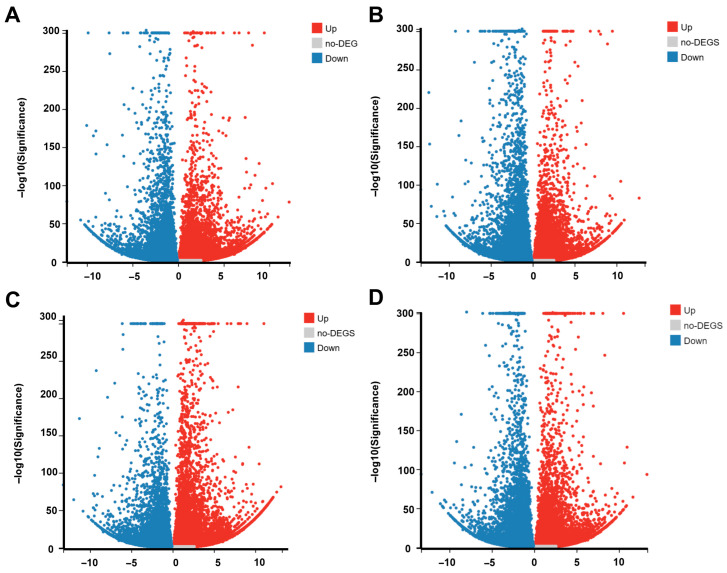
DEGs volcano map of each experimental group compared with the control group (pH 5.5). (**A**) pH 4.0 for 3 h and untreated. (**B**) pH 4.0 for 6 h and untreated. (**C**) pH 8.5 for 3 h and untreated. (**D**) pH 8.5 for 6 h and untreated.

Compared with unigene group A, group B had 29,139 DEGs (14,669 upregulated and 14,470 down-regulated), group C had 32,366 DEGs (15,909 upregulated and 16,457 down-regulated), group D had 36,706 DEGs (21,108 upregulated and 15,598 down-regulated), and group E had 31,228 DEGs (15,505 upregulated and 15,723 down-regulated; Figure 1A). The number of upregulated genes relative to the control was largest after 3 h alkali treatment (Figure 1B). In total, 75,171 DEGs were common to the five groups; 630 and 635 genes were significantly expressed only after 3 h and 6 h acid treatment, respectively, and 4200 and 605 genes were significantly expressed only after 3 h and 6 h alkali treatment, respectively. Among DEGs, 10,281 genes were differentially expressed in all comparison groups; 3187 and 3943 genes were differentially expressed after 3 h and 6 h acid treatment, respectively, and 10,120 and 3744 genes were differentially expressed after 3 h and 6 h alkali treatment, respectively (Figure 1C,D). Volcano maps of the relative differential gene expressions are provided in Figure 2D. All upregulated and down-regulated DEGs are listed in Supplementary Files S1–S8.

### 2.2. Differential Gene Expression Under Acid and Alkali Treatments

**Figure 3:**
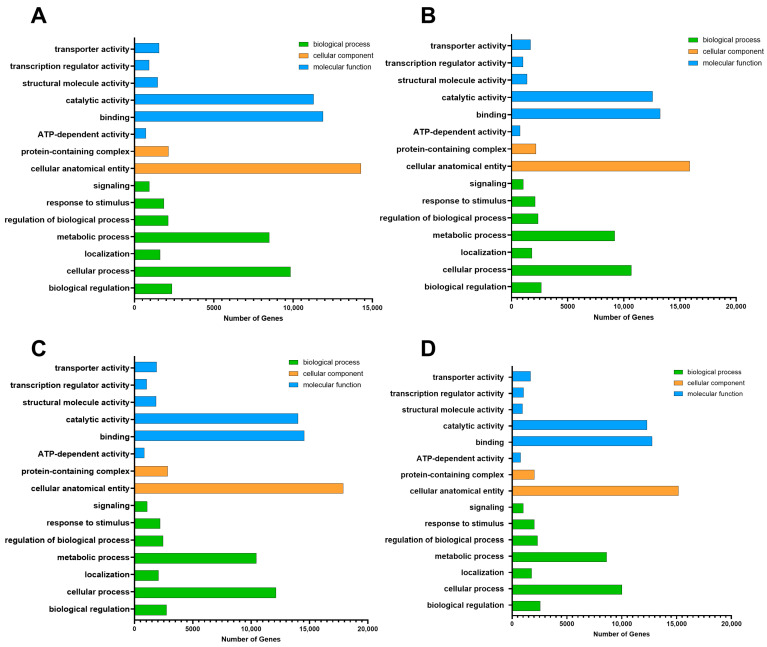
GO annotation diagram of DEGs in each experimental group compared with the control group. (**A**) pH 4.0 for 3 h versus untreated. (**B**) pH 4.0 for 6 h versus untreated. (**C**) pH 8.5 for 3 h versus untreated. (**D**) pH 8.5 for 6 h versus untreated.

**Figure 4:**
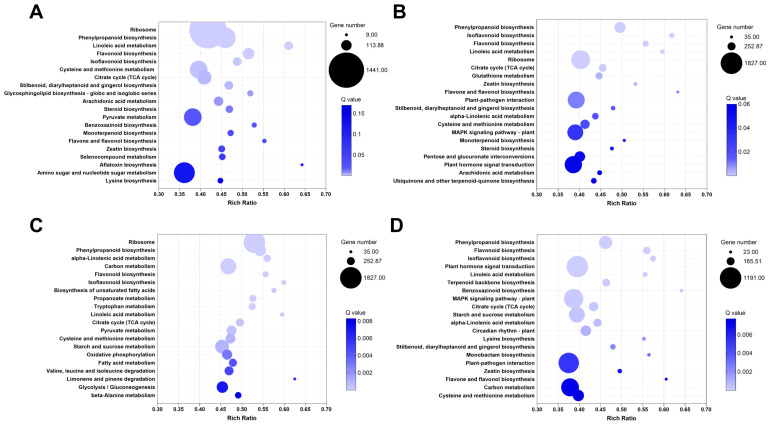
KEGG pathways of DEGs in each experimental group compared with the control group. (**A**) pH 4.0 treated 3 h versus untreated. (**B**) pH 4.0 treated 6 h versus untreated. (**C**) pH 8.5 for 3 h versus untreated. (**D**) pH 8.5 for 6 h versus untreated.

**Figure 5:**
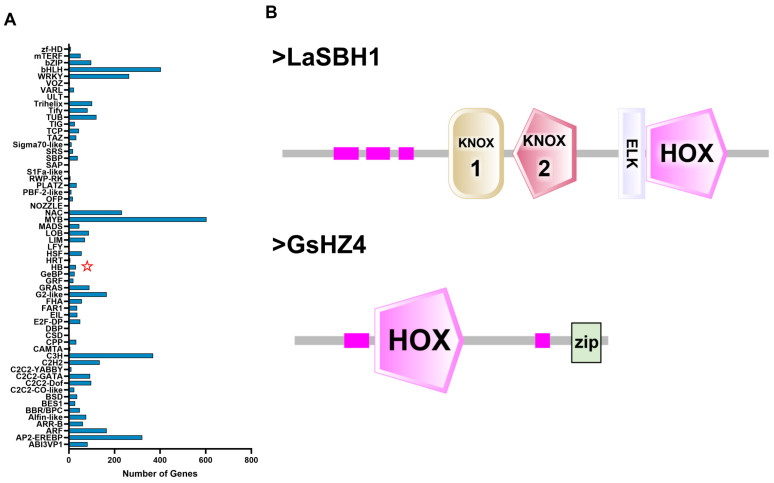
Transcription factor families and domains analysis in lupinus. (**A**) Transcription factors annotation and classification in all DEGs in lupinus. The HB family is marked with a star. (**B**) Conservative domain comparison between *LaSBH1* and *GsHZ4*. The pink squares represent the conserved motifs.

Gene annotation using the GO database revealed little difference in DEG classification after acid or alkali treatment for 3 h or 6 h. Under acid and alkaline treatments, DEGs participated in processes related to cellular anatomical entities, binding, and catalytic activity, as well as cellular and metabolic processes. The number of DEGs in each of these categories differed between treatment types. Under 3 h and 6 h acid treatment, most DEGs in the biological process category were involved in cell and metabolic processes (Figure 3A,B). Most of those in the cellular component category were related to cellular anatomical entities, and most in the molecular function category were involved in binding and catalytic activity (Figure 3C,D). KEGG terms for corresponding pathways are shown in Figure 4A–D. Under 3 h acid or alkaline stress, most upregulated genes participated in ribosome pathways; under 6 h acid or alkaline treatment, genes involved in plant–pathogen interactions and the MAPK signaling pathway were upregulated. The expression of lupinus genes related to the synthesis of organic matter changed first at 3 h, and those related to the plant hormone and signal transduction response changed at 6 h. The largest number of transcription factors was from the MYB family. We found that there are no HD-Zip family genes in lupinus genomes, but in HB, MYB, C3H, AP2, bHLH, and other transcription factor genes (Supplementary File S9 and Figure 5A). There are a few HB genes in lupinus, and we chose *LaSBH1* as an example to show the conserved domains in them. Both *LaSBH1* and *GsHZ4* have the same HOX domain, but *GsHZ4* from the HD-ZIP family has the special ‘Zip’ domain at the end of HOX domain (Figure 5B). So, it was predicted that the mission of the ‘Zip’ domain in key genes may cause a tolerance reduction in lupinus.

### 2.3. GsHZ4 Overexpression in Lupinus Roots Improved Alkali Stress Tolerance

**Figure 6:**
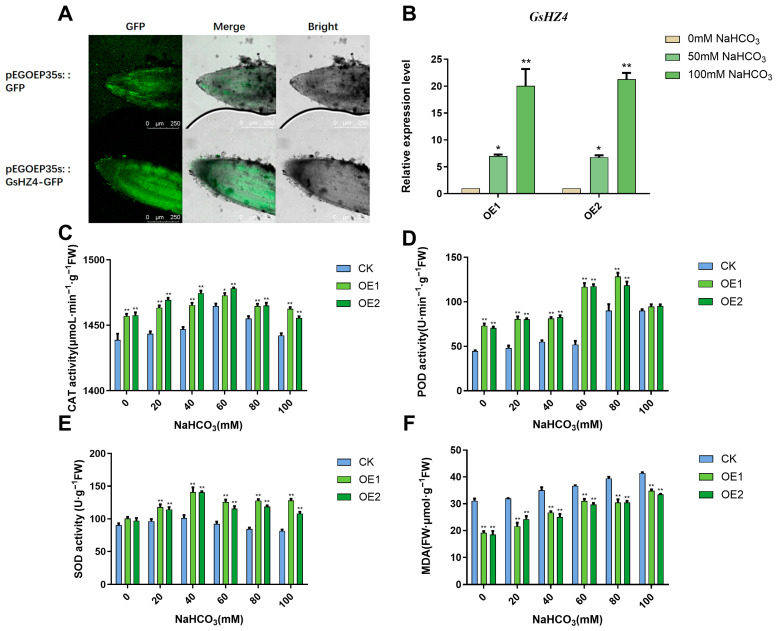
Overexpression of *GsHZ4* in lupinus hairy roots can improve alkaline resistance. (**A**) Laser confocal microscopy was used to detect the GFP signal in the root tips of new lupinus roots and the signal containing *GsHZ4*-GFP was more strongly expressed in the root tips. (**B**) The expression level of the *GsHZ4* gene in transgenic roots treated with 0 mM, 50 mM, and 100 mM NaHCO_3_. (**C**) CAT enzyme activity. (**D**) POD enzyme activity. (**E**) SOD enzyme activity. (**F**) MDA content. For (**B**–**F**), a significant difference was calculated by Student’s *t*-test (* *p* < 0.05, ** *p* < 0.01).

We separately transformed the GFP empty vector and *GsHZ4*-GFP into the root tips of mung bean, this signal was distributed evenly in multiple areas of the root tips, indicating that *GsHZ4*-GFP could be stably expressed in the root tips (Figure 6A). The relative expression of *GsHZ4* increased with 0 mM, 50 mM, and 100 mM NaHCO_3_ treatment (Figure 6B). The experiments proved that lupinus could generate transgenic hairy roots, and that wild soybean genes could be expressed in response to alkali treatment. Relative to those in WT roots, the CAT and POD activity levels were significantly higher in overexpressed plants before treatment; CAT activity levels were higher under 20 mM, 40 mM, and 60 mM NaHCO_3_ treatment (Figure 6C), and POD activity levels were higher at all concentrations less than 100 mM (Figure 6D). The SOD content of overexpressed plants was significantly greater than that of the WT under 40 mM, 60 mM, 80 mM, and 100 mM NaHCO_3_ treatment (Figure 6E). The MDA content was significantly less, indicating less membrane damage in the overexpressed plants than in the WT under all treatments (Figure 6F). These results indicate that *GsHZ4* improved antioxidant enzyme activity and maintained cell membrane permeability in lupinus. The highest alkali tolerance threshold for transgenic plants was about 100 mM.

### 2.4. GsHZ4 Overexpression Increased Plant Growth Under Alkali Stress

**Figure 7:**
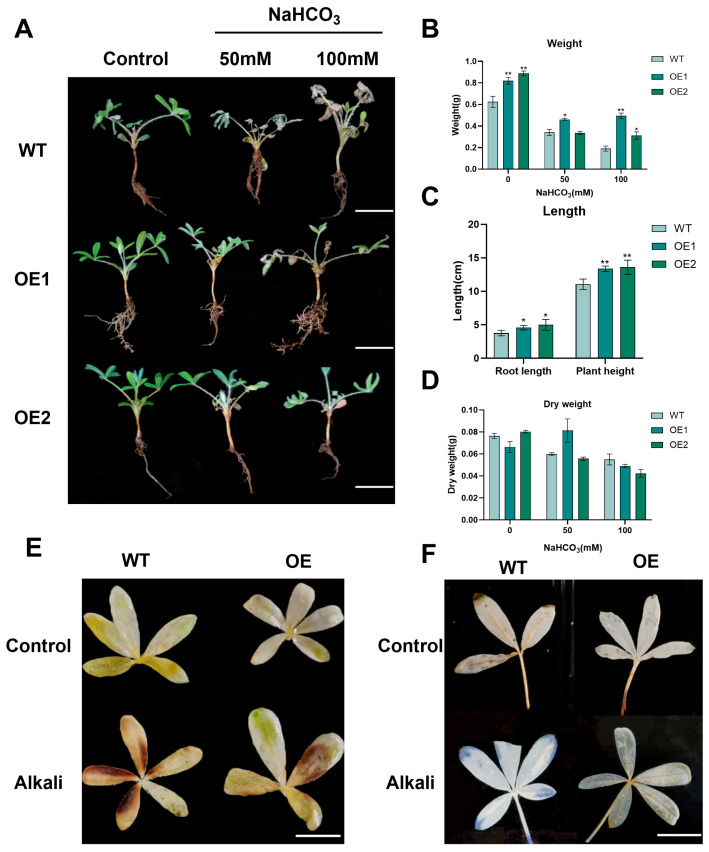
Phenotype observation of lupinus seedlings. (**A**) Plants (one control group and two groups of overexpressed plants) treated with 0 mM, 50 mM, and 100 mM NaHCO_3_. Bar = 2 cm. (**B**) Determination of fresh plant weight in each group. (**C**) The dry weight of each group was determined. (**D**) The root length of each group was measured. (**E**) DAB staining experiments on the leaves of the control group and the experimental group under the treatment of 0 mM and 100 mM NaHCO_3_. The brown part represents oxidative damage. Bar = 1 cm. (**F**) NBT staining of leaves in the control group and the experimental group under the treatment of 0 mM and 100 mM NaHCO_3_. The blue part represents oxidative damage. Bar = 1 cm. For (**B**–**D**), a significant difference was calculated by Student’s *t*-test (* *p* < 0.05, ** *p* < 0.01).

Under control conditions, both the overexpression and control group plants exhibited comparable, normal growth phenotypes. The WT strains showed leaf wilting, yellowing, curling, and shrinking after 48 h treatment with 50 mM NaHCO_3_, whereas the overexpressed plants showed no obvious change. After 48 h treatment with 100 mM NaHCO_3_, the WT plants wilted completely, with thin stems and curled, wrinkled leaves; the overexpressed plants showed some leaf yellowing but grew normally (Figure 7A). The weight and root length of the overexpressed plants were significantly greater than those of the WT plants (Figure 7B–D). DAB and NBT staining were similar in untreated overexpressed and WT plant leaves, but weaker in overexpressed than in WT leaves, reflecting lesser H_2_O_2_ and O_2_^−^ contents, under 100 mM NaHCO_3_ (Figure 7E,F). Thus, *GsHZ4* overexpression improved the alkali tolerance of lupinus roots, leading to improved oxidation resistance and tolerance of the plants’ aboveground parts.

### 2.5. GsHZ4 Improved Plant Alkaline Tolerance by Regulating Alkali Stress-Related Genes

**Figure 8:**
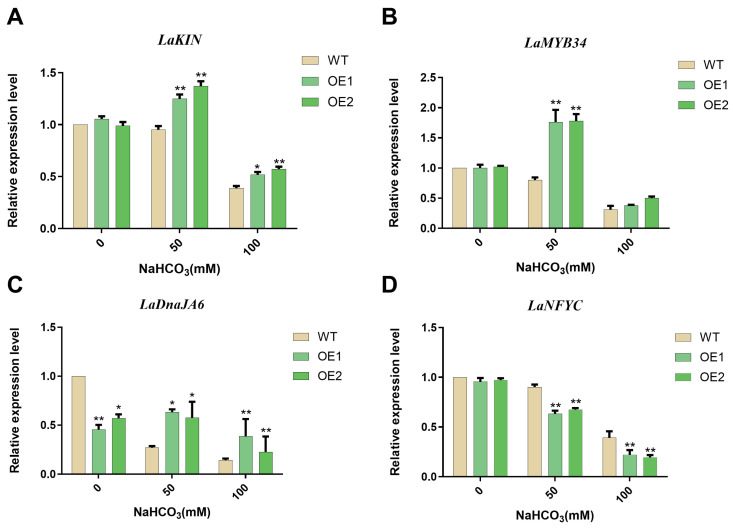
Relative expression levels of alkali stress related marker genes in WT and two groups of overexpressed plants treated with 0 mM, 50 mM, and 100 mM NaHCO_3_. (**A**) *LaKIN* (**B**) *LaMYB34* (**C**) *LaDanJA6* (**D**) *LaNFYC*. For A–D, a significant difference was calculated by Student’s *t*-test (* *p* < 0.05, ** *p* < 0.01).

Alkali stress-related genes were detected in overexpressed plants to predict the alkali resistance regulatory pathway of *GsHZ4* [20]. In untreated plants, the relative expression of all genes was significantly lower than in the WT. *LaKIN* is a gene responsible for sustained responses to alkali stress. *LaMYB34* is a transcription factor gene that responds to alkali stress [9]. Under 50 mM NaHCO_3_ treatment, the relative expression of *LaKIN* and *LaMYB34* in *GsHZ4*-overexpressing lines was greater than in WT plants (Figure 8A,B). The relative expression of *LaKIN* and *LaMYB34* was significantly decreased in overexpressed lines relative to that in WT plants under 100 mM NaHCO_3_ treatment. The expression level of *LaDnaJA6* (a downstream functional gene) decreased with increasing alkaline concentration in WT, while it exhibited an upward trend in overexpression lines (Figure 8C). The expression level of *LaNFYC* (responsible for sustained responses to alkali stress) generally exhibited a downward trend (Figure 8D). These findings suggest that *GsHZ4* regulates the *LaKIN*-based response to alkali stress. In addition, *LaMYB34* may be located in the same pathway as *GsHZ4*.

**Figure 9:**
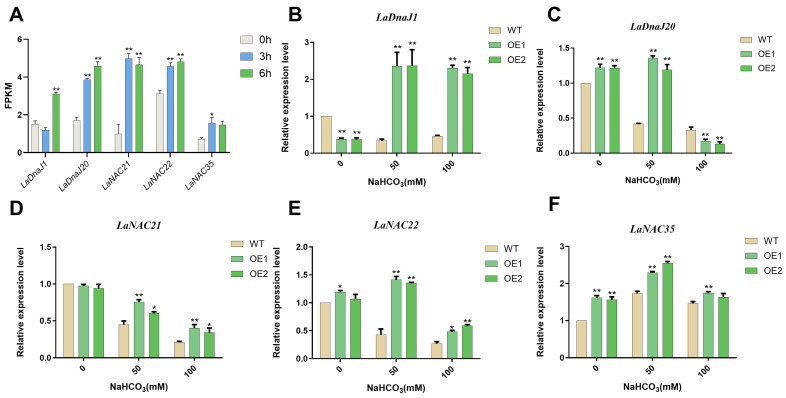
Relative expression levels of alkali stress-related genes from the RNA-seq data. WT and two groups of overexpressed plants were treated with 0 mM, 50 mM, and 100 mM NaHCO_3_. (**A**) FPKM values of genes related to alkali stress (**B**) *LaDnaJ1*, (**C**) *LaDnaJ20*, (**D**) *LaNAC21*, (**E**) *LaNAC22*, and (**F**) *LaNAC35*. For (**A**–**F**) a significant difference was calculated by Student’s *t*-test (* *p* < 0.05, ** *p* < 0.01).

The alkaline response-related genes were screened out from the transcriptome sequencing results (Figure 9A). The expression level of *LaDNAJ1* was relatively low before any processing; its expression was significantly greater in overexpressed plants than in the WT under 50 and 100 mM NaHCO_3_ treatment, and *LaDnaJ20* expression was significantly greater in overexpressed plants than in the WT under 0 and 50 mM NaHCO_3_ treatment (Figure 9B,C). However, the expression levels of *LaDnaJ20*, *LaNAC22*, and *LaNAC35* were significantly lower in *GsHZ4*-overexpressing lines under 100 mM NaHCO_3_ treatment. The relative expression of *LaNAC21* was significantly lower in overexpressed than in WT plants under all treatments (Figure 9D). The relative expression of *LaNAC22* was greater in overexpressed than in WT plants under 50 mM NaHCO_3_ treatment (Figure 9E). The relative expression of *LaNAC35* was higher in untreated overexpressed plants than in the WT, but significantly lower than in the WT under all treatments (Figure 9F). These results suggest that *GsHZ4* transfer improved plant alkaline tolerance by regulating the expression of related genes (*LaDnaJ1*, *LaDnaJ20*, and *LaNAC22*) under alkali stress.

### 2.6. GsHZ4 Was Induced by IAA in Lupinus Roots and Was Slightly Responsive to ABA, JA, and GA

**Figure 10:**
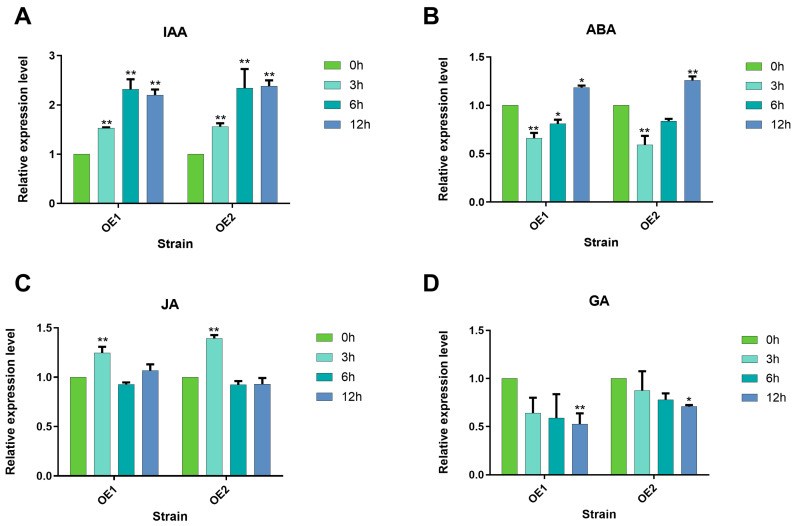
The relative expression of *GsHZ4* in the roots of lupinus seedlings (WT and *pGsHZ4::GsHZ4 overexpression lines*) treated with different hormones at 0 h, 3 h, 6 h, and 12 h was measured in OE groups. (**A**) 4 mg/L ABA (**B**) 5 μM IAA (**C**) 10 μM JA (**D**) 0.5 μM GA. For (**A**–**D**), a significant difference was calculated by Student’s *t*-test (* *p* < 0.05, ** *p* < 0.01).

*GsHZ4* was significantly upregulated by IAA (Figure 10A–D). Under ABA (abscisic acid) treatment, its expression was down-regulated at 6 h and upregulated at 12 h. Under JA (jasmonic acid) treatment, *GsHZ4* expression was significantly upregulated at 3 h. *GsHZ4* expression was significantly down-regulated after 12 h of GA (gibberellin) treatment. These results indicate that *GsHZ4* in lupinus was induced mainly by IAA and regulated by ABA and was involved mainly in the IAA and ABA signaling pathways. In hormone treatment experiments, *GsHZ4* is initiated by its own promoter in roots of lupinus plants.

## 3. Discussion

### 3.1. Transcriptome Sequencing Analysis of Acid/Alkali Response Genes

Transcriptome sequencing is a high-throughput sequencing method applied in research conducted under defined environmental conditions. It has been used to examine plants’ salt stress tolerance, a vital step in efforts to breed salt-resistant crops [21]. Different pH treatments can reveal differentially expressed genes in plants. For example, in tomato roots exposed to varying pH conditions, transpiration rates and stomatal conductance show distinct alterations [22]. Given the limited research progress reported on lupinus, we performed transcriptome sequencing on lupinus under different pH conditions to facilitate the identification of biologically significant insights. In this study, transcriptome sequencing revealed that lupinus lacks HD-Zip transcription factors, a finding that may explain its sensitivity to alkaline stress. The use of the *Agrobacterium rhizogenes*-mediated transformation and *GsHZ4* to obtain chimeric lupinus plants with improved alkaline tolerance is an innovative aspect of this research. *GsHZ4* was successfully transferred into lupinus hairy roots via *Agrobacterium rhizogenes* and expressed. The qPCR result analysis indicated that *GsHZ4* increased the expression levels of alkali stress-related genes (*LaKIN*, *LaMYB34*, *LaDnaJ1*, *LaDnaJ20*, and *LaNAC21*) which differed under alkali stress (Figure 8 and Figure 9). These results indicate the feasibility of using various stress resistance regulation models from wild soybean to improve the ornamental characteristics of lupinus.

### 3.2. The Role of HD-ZIP Family Genes in Alkali Stress Response

The HD-ZIP (homeodomain-leucine zipper) family of transcription factors plays a pivotal role in regulating plant growth, developmental processes, and stress responses, particularly under alkali stress conditions. Alkali stress, characterized by elevated pH levels and excessive sodium carbonate/bicarbonate concentrations, induces significant osmotic and ionic toxicity in plants, resulting in growth retardation and cellular damage [23]. Emerging evidence indicates that HD-ZIP genes participate in plant adaptation to alkaline environments through the regulation of stress-responsive gene networks and maintenance of ion homeostasis. In *Arabidopsis thaliana*, for example, *AtHB7* (a member of the HD-ZIP I subfamily) has been shown to improve alkali tolerance by modulating ABA-mediated signaling cascades and osmotic adjustment mechanisms. Similarly, Medicago truncatula exhibits upregulation of *MtHB2* under alkali stress conditions, implying its functional involvement in stress adaptation [24]. Notably, recent studies have revealed that the HD-ZIP family contributes to the regulation of plant height in *Lagerstroemia* species and mediates stress response mechanisms. The evolutionary conservation of structural domains among HD-ZIP members, especially in their DNA-binding homeodomains and leucine zipper motifs, suggests the existence of conserved regulatory mechanisms across diverse plant species [25]. In light of increasing global soil alkalization problems, elucidating the molecular mechanisms underlying HD-ZIP-mediated alkali stress responses could provide valuable insights for developing stress-tolerant lupinus cultivars, thereby facilitating their cultivation in marginal agricultural lands. This study, based on the results of transcriptome sequencing, found that the HD-ZIP family genes were absent in the transcriptome of lupinus. However, as both belong to the legume family, there are significant differences in their alkaline tolerance between lupinus, wild soybean, and soybean. Since previous experiments demonstrated that *GsHZ4* enhances alkali tolerance in *Arabidopsis thaliana* [9], we initiated this study to introduce this exogenous gene into lupinus.

### 3.3. Mechanism of Alkali Stress Regulation by the Transcription Factor GsHZ4

Among the DEGs identified in this study, we found HB transcription factors that lack a zip domain (Figure 5). Gene Ontology analysis indicated that the products of these HB family genes are involved in cellular component organization and biological processes. *LaKIN1* expression was found to be increased in *GsHZ4*-overexpressing *Arabidopsis thaliana*, suggesting that *GsHZ4* positively regulates this expression and participates in the response to bicarbonate stress [9]. MYB is involved in the regulation of a variety of biological processes. *GmMYB14* may be involved in plant responses and resistance to environmental stresses via the regulation of related gene expression [26]. DnaJA proteins act as molecular chaperones related to environmental stresses in legumes. For example, *GmDnaJA6* is a candidate regulator of salt-alkali stress resistance that confers increased tolerance of this stress in soybeans overexpressing it [2]. The expression levels of *GmDnaJA9* and *GmDnaJA13* are higher under salt and alkali stresses, especially after 6 and 12 h, compared with the control conditions. These findings suggest that wild soybean genes can regulate lupinus genes and improve the stress tolerance of lupinus. However, we found that *GsHZ4* overexpression in lupinus inhibited the expression of genes such as *LaDnaJA6*, *LaNFYC*, *LaNAC21*, and *LaNAC35* (Figure 8 and Figure 9). These genes may be located in different pathways. *GmNF-YC14*, which encodes an NF-YC member in soybean, forms heterotrimers with *GmNF-YA16* and *GmNF-YB2* to activate the *GMPYR1*-mediated ABA signaling pathway, thereby regulating soybean tolerance to drought and salt stresses [27]. These results suggest that *GsHZ4* may function through specific regulatory pathways and protein interactions, exerting its influence via coordinated action within the broader regulatory network. The diverse functions of *GsHZ4* in lupinus need to be studied further.

### 3.4. Hormone-Mediated Alkali Stress Tolerance

Hormones play important regulatory roles in plant growth. IAA induces crop root development [28]. Auxin regulates the expression of HD-Zip genes via the elegantly short TIR1/AFB signal transduction pathway [29]. Several auxin response elements (AuxREs) have been recognized in the promoters of wheat HD-Zip subfamily genes, with the upregulation of the transcript levels of *PIN1*, *PIN3*, and *PIN4*. Under saline conditions, auxin affects the direction and depth of root growth, helping plants to better absorb water and nutrients and adapt [30]. *AtHB2* encodes an HD-Zip transcription factor and functions as an auxin responsive gene in Arabidopsis [31]. Some HD-Zip genes are involved in the ABA-mediated pathway [32]. While no prior studies have reported the response of *GsHZ4* to IAA induction, its functional homolog, *GhHB12*, is known to be auxin-inducible. *GhHB12* has been implicated in regulating plant height in cotton by modulating auxin signal transduction and cell wall expansion [33]. In this study, the roots of untreated plants grew longer than those of the control, likely due to the participation of *GsHZ4* promoter in the IAA regulatory pathway, resulting in NaHCO_3_ and alkaline stress tolerance. NaHCO_3_ resistance processes may share the same pathway with IAA and ABA hormone regulation. In this project, we found that these response mechanisms could not be activated in the absence of HD-Zip genes, which may be the root cause of the difference in stress tolerance between wild soybean and lupinus. Further study of the molecular mechanisms of *GsHZ4*’s activity in lupinus is needed. 

## 4. Materials and Methods

### 4.1. Plant Material and Culture Conditions

The experiment was conducted at the Horticulture Experimental Center of Northeast Agricultural University, situated at 45°44′28″ N and 126°43′16″ E. The lupinus seeds were purchased from the commercial supplier ‘Hua You Xiu’. Seedlings were initially cultured in nutrient pots filled with a soil mixture (nutrient soil:vermiculite = 1:1). After the development of the third pair of leaves, they were transferred to a hydroponic system with pH 5.5 Hoagland solution. The bowls were maintained at a controlled temperature of 22–25 °C under a 16 h light/8 h dark photoperiod until the third pair of palmate compound leaves had fully developed. The seedlings were transferred to Hoagland solution for NaHCO_3_ stress and hormone treatment. Wild soybean seeds were surface-sterilized by briefly soaking in concentrated sulfuric acid for 5 min, followed by rinsing with distilled water 5 times. The sample was placed in an incubator at 28 °C for 48 h and cultured in 1/4 Hoagland medium (pH 5.8) for 21 days. The temperature was 26-30 °C with 16 h light/8 h dark conditions [34].

### 4.2. Transcriptome Sequencing Sampling and Detection Methods

Lupinus plants were cultured in pH 5.5 Hoagland solution as a control (group A/a). Other lupinus plants were treated in pH 4.0 and pH 8.2 Hoagland solution for 3 h (group B/b and D/d) and 6 h (group C/c and E/e). Root tips were frozen with liquid nitrogen and stored in the -80 °C refrigerator. Each group of samples was taken 3 times. A total of 15 samples were sent to the BGI for transcriptome sequencing. The data were uploaded to the NCBI SRA database (SUB14514279).

### 4.3. Differential Expression Genes Analysis

SOAPnuke (v1.6.5), developed by the BGI (Beijing Genomics Institution), was used as a filter software to remove reads containing joints; reads with unknown base N content greater than 1% were removed. Low-quality reads were removed. The filtered ‘Clean Reads’ are saved in FASTQ format. Bowtie2 (https://bowtie-bio.sourceforge.net/bowtie2/index.shtml, accessed on 2 November 2025), a memory-efficient short-read aligner utilizing the Burrows–Wheeler transform and FM-indexing algorithm, was employed to perform sequence alignment and quantify gene/transcript expression levels (in FPKM/RPKM/TPM) by mapping the processed sequencing reads against the reference genome/transcriptome. This alignment-based quantification enabled comparative analysis of sequence utilization efficiency across different assembly strategies. Trinity (v2.13.2) was used for de novo assembly of clean reads (removing PCR duplicates to improve assembly efficiency). CD-HIT (CD-HIT: 4.6) was used for clustering to remove redundancy to obtain the unigenes. Unigenes were compared to the SwissProt database and Pfam database. TransDecoder.Predict (v5.5.0) was used to predict the CDS structures, such as 3’ UTR, 5’ UTR, CDS, and mRNA, etc. Venn diagrams were drawn using Venn (version 1.7). The volcano map of the DEGs was created using Sangerbox [35].

### 4.4. GO Annotation and KEGG Analysis

All unigene sequences were aligned to the NR (http://ftp.ncbi.nlm.nih.gov/blast/db, accessed on 2 November 2025), NT (http://ftp.ncbi.nlm.nih.gov/blast/db, accessed on 2 November 2025), Uniprot (https://www.uniprot.org, accessed on 2 November 2025), KEGG (https://www.kegg.jp, accessed on 2 November 2025), GO (http://geneontology.org, accessed on 2 November 2025), and COG (clusters of orthologous groups) (https://www.ncbi.nlm.nih.gov/COG/, accessed on 2 November 2025) databases. The GO (Gene Ontology) database consists of three branches: molecular functions, biological processes, and cellular components which were used to describe the function of gene products. According to KEGG annotation, the differential expressed genes were classified into different biological pathways. Generally, the Q value ≤ 0.05 was considered as significant enrichment. Trinity assembly of quality statistics of filtered reads is shown in Table 1. Results of the reference genome alignment are shown in Table 2. The annotated number and ratio of the unigenes in various databases is shown in Table 3. The annotated number and ratio of the unigenes in various databases is shown in Table 4.

### 4.5. Determination of Positive Transgenic Hairy Roots and Physiological Signs

Gene cloning primers are listed in Table 5. The empty vector pEGOEP35S (p35S)-GFP (Biorun, Wuhan, China) carries the green fluorescent protein (GFP) gene. The p35S-GFP construct was generated by replacing the GUS coding sequence in the pBI121 backbone with a functional GFP gene, thereby placing GFP expression under the control of the CaMV 35S promoter. Protoplasts were observed under incident-light fluorescence microscopy 24 h after electroporation, with 20–60% of protoplasts exhibiting intense green fluorescence upon excitation with blue light (450–490 nm). Agrobacterium rhizogenes strain K599 harboring either pEGOEP35S::*GsHZ4*-GFP or the empty pEGOEP35S::GFP plasmid was cultured at 28 °C. A 5 mL aliquot of LB liquid medium supplemented with kanamycin (50 μg/mL) was dispensed into a sterile 15 mL polypropylene tube. Single colonies were inoculated into the medium and incubated at 28 °C with shaking at 180 rpm for 48 h. The bacterial culture was then collected and centrifuged. After discarding the supernatant, the pellet was resuspended to a final volume of 400 μL, and the suspension was spread onto LB solid medium followed by incubation at 28 °C for 2 days until dense Agrobacterium growth was observed. After lupinus seed germination, roots were excised at a 45° angle approximately 1 cm below the cotyledons. The cut surfaces of both control and experimental seedlings were thoroughly coated with the corresponding Agrobacterium suspension and placed on moist filter paper in Petri dishes. All seedlings were maintained in an incubator at 28 °C for 48 h, after which they were transferred and planted in pre-soaked vermiculite. The vector was transferred into lupinus to determine the phenotype, activity of stress-related enzymes, and marker genes. RT-PCR identification is shown in Figure S1. In RT-qPCR experiments, the 35S promoter on pBI121-35S::*GsHZ4* was replaced by a promoter of *GsHZ4* in transgenic plants to detect the expression level of *GsHZ4* under hormone and alkaline treatments.

After the seedlings had developed three palmate compound leaves, root tips from transgenic (positive) and control plants were excised. These samples were then observed and imaged using a confocal laser scanning microscope (Leica, Wetzlar, Hessen, Germany) and photographed. Transgenic overexpression lines were identified and confirmed by quantifying *GsHZ4* expression levels using qPCR. The expression of *GsHZ4*, being an exogenous gene, was undetectable in wild-type (WT) plants. For qPCR analysis, the expression level of *GsHZ4* in each treated transgenic sample was normalized to an internal reference gene (*Ubiquitin*) and was calculated relative to its expression in the untreated transgenic control (set as 1.0) using the 2^-ΔΔCt^ method. Transgenic and non-transgenic (WT) plants were treated with NaHCO_3_ solutions at concentrations of 0 mM, 20 mM, 40 mM, 60 mM, 80 mM, and 100 mM. The activities of POD, CAT, and SOD, as well as the MDA content, were measured using commercial assay kits according to the manufacturers’ protocols. Kits (POD: G0107W; CAT: G0105W, SOD: G0101W; MDA: G0109W) were from Geruisi Biotechnology Co., LTD., Suzhou, China.

### 4.6. Root Phenotype Analysis

To generate transgenic hairy roots, *Agrobacterium rhizogenes* strain K599 harboring the pEGOEP35S::*GsHZ4*-GFP plasmid was used for infectionHairy roots induced by the empty vector (K599 without the *GsHZ4* insert) served as the control group. Plants were treated with 0 mM, 50 mM or 100 mM NaHCO_3_ solution to observe phenotypic responses [20].

### 4.7. Diaminobenzidine (DAB) Staining

The DAB staining experiment can indicate the H_2_O_2_ content in plant tissues, and the experiment was conducted by using DAB powder (Biotopped, Beijing, China) to prepare a staining solution. We dissolved 0.1 g DAB powder in a brown bottle with 45 mL distilled water (prepared away from light). Then, we adjusted the pH to 5.7, and added distilled water to 50 mL. The leaves were placed in DAB dyeing solution for vacuum treatment and stained for 18 h. The dyed leaves were soaked in anhydrous ethanol, and anhydrous ethanol was added to them appropriately during the boiling process to prevent drying. They were removed thoroughly after decolorization and placed on a glass slide dripping with glycerin. They were then observed and recorded by a digital camera [36].

### 4.8. Nitrotetrazolium Chloride Blue Solution (NBT) Staining

NBT staining was used to detect the content of superoxide anion in plant tissue. Experiments were performed by using NBT powder (Biotopped, Beijing, China) to prepare a staining solution. NaH_2_PO_4_ buffer and Na_2_HPO_4_ buffer were configured. A total of 1.6 mL NaH_2_PO_4_ buffer and 8.4 mL Na_2_HPO_4_ buffer were mixed and distilled water was added to 200 mL. The pH was adjusted to 7.5. Then, 0.1 g of NBT powder was dissolved in 50 mL of the phosphate buffer in a brown bottle. Leaves were immersed in the NBT solution and incubated in the dark for 18 h. The subsequent steps of decolorization (in anhydrous ethanol), mounting (in glycerol), and image capture were performed as described for DAB staining. They were then observed and recorded by a digital camera [36].

### 4.9. Determination of Relative Expression Levels of Key Genes

Total RNA was extracted from root tips of both wild-type (WT) and transgenic lupinus seedlings treated with 0 mM, 50 mM or 100 mM NaHCO_3_, using a TransZol Up kit (ET111-01-V2; TransGen Biotech, Beijing, China). Using the extracted RNA as template, cDNA was synthesized by reverse transcription with a SPARKscript RT Plus kit (with gDNA eraser; AG0304-B, Sparkjade, Shandong, China). The relative expression levels of *GsHZ4* and key alkali stress-related genes—*LaKIN*, *LaDnaJA6*, *LaNFYC*, *LaMYB34*, *LaDnaJ1*, *LaDnaJ20*, *LaNAC21*, *LaNAC22*, and *LaNAC35*—were determined by quantitative PCR (qPCR; AH0104-B, Sparkjade, Shandong, China). The relative expression of *GsHZ4* was also measured in roots of WT and transgenic lupinus seedlings treated with 4 mg/L ABA, 5 μM IAA, 0.5 μM GA, or 10 μM JA for 0 h, 3 h, 6 h, and 12 h, respectively [2]. Because *GsHZ4* is an exogenous gene, its expression was undetectable in wild-type lupinus. Therefore, in the results, the expression level of *GsHZ4* was compared to that in the corresponding untreated overexpression plants (0 mM NaHCO_3_ or 0 h hormone treatment) as the control. All data were calculated using the 2^−ΔΔCT^ method. The sequences of all primers used are provided in Table 5.

### 4.10. Statistical Analysis

Means and standard deviations of all study variables were calculated and compared between samples using Student’s *t* test. For multiple comparisons, two-factor analysis of variance and post hoc Tukey testing were performed using GraphPad Prism software 10.4.1. In this project, ‘*’ represented *p* < 0.05; ‘**’ represented *p* < 0.01.

## 5. Conclusions

Lupinus is a legume that requires an acidic environment and is highly sensitive to alkaline stress. Wild soybean, also part of the legume family, has strong alkaline resistance. In this study, transcriptome sequencing was used to obtain different acid-base response genes in lupinus and GO annotation; KEGG pathway analysis of the genes showed that the acid and alkaline response pathways were completely different in lupinus. Comparative analysis revealed that lupinus lacks homologs of key alkali-tolerance genes present in wild soybean, such as those encoding HD-Zip transcription factors. *GsHZ4*, an alkali tolerance-related HD-Zip gene in wild soybean that is absent in lupinus, was transferred into hairy lupinus roots and was expressed in the root tips. *GsHZ4* expression in lupinus was induced by IAA via its own promoter, and lupinus plant tolerance of alkali stress was improved by the regulation of lupinus stress-related gene expression. This study demonstrates that the abundant resistance-gene resources in wild soybean can be utilized to enhance abiotic stress tolerance in lupinus, offering a viable strategy for expanding its cultivation.

## Figures and Tables

**Table 1:** Results of Trinity assembly.

Unigene	Total Length	Average Length	N50	GC%
104,353	166,491,728 bp	1595 bp	2183 bp	40.45%

**Table 2:** Quality statistics of filtered reads.

Sample	Total Raw Reads (M)	Total Clean Reads (M)	Total Clean Bases (Gb)	Clean Reads Q20 (%)	Clean Reads Q30 (%)	Clean Reads Ratio (%)
A1	45.57	42.35	6.35	97.42	93.19	92.93
A2	47.33	43.43	6.52	96.75	92.16	91.78
A3	45.57	42.64	6.4	96.75	92.12	93.56
B1	45.57	42.72	6.41	97.39	93.12	93.74
B2	47.33	42.27	6.34	96.72	92.07	89.31
B3	45.57	42.68	6.4	96.53	91.65	93.64
C1	45.57	42.68	6.4	97.42	93.2	93.65
C2	45.57	42.3	6.34	96.84	92.32	92.81
C3	45.57	42.43	6.36	96.67	91.98	93.09
D1	45.57	42.04	6.31	96.75	92.11	92.24
D2	45.57	42.36	6.35	96.58	91.77	92.95
D3	47.33	42.93	6.44	96.66	91.97	90.7
E1	47.33	43.24	6.49	96.66	91.96	91.37
E2	45.57	42.15	6.32	96.73	92	92.48
E3	47.33	42.28	6.34	96.81	92.29	89.34

**Table 3:** Result of reference genome alignment.

Sample	Total Clean Reads (M)	Total Mapping (%)	Uniquely Mapping (%)
A1	42.35	86.73	17.58
A2	43.43	87.39	17.72
A3	42.64	87.38	17.73
B1	42.72	86.51	17.88
B2	42.27	85.02	17.49
B3	42.68	87.02	18.1
C1	42.68	87.35	18.27
C2	42.3	87.03	18.42
C3	42.43	87.46	18.43
D1	42.04	84.99	17.81
D2	42.36	85.43	17.98
D3	42.93	84.19	17.7
E1	43.24	86.77	17.78
E2	42.15	86.38	17.71
E3	42.28	85.94	17.45

**Table 4:** Annotated number and ratio of the unigenes in various databases.

Databases	Number of Unigenes	Ratio (%)
All	104,353	100.00%
NR	94,646	90.07%
NT	92,967	89.09%
Swiss Prot	75,069	71.94%
KOG	77,746	74.50%
KEGG	76,181	73.00%
GO	77,940	74.69%
Pfram	76,334	73.15%

**Table 5:** Primer sequences for gene cloning and qPCR analysis.

Primer Name	Primer Sequences (5′-3′)
*GsHZ4*-F	CCACCTTTCCAAGACCAC
*GsHZ4*-R	CTCCATTGCCAGCCTATC
*Ubiquitin*-F	GGCAAGACCATCACTCTCGA
*Ubiquitin*-R	ACCTCAAGGGTGATGGTCT
*LaKIN*-F	AAGCTTCAACCTCACGAGCC
*LaKIN*-R	CAATTCCATAACTCCTGCGTGG
*LaMYB34*-F	GCAAAGTTTGGCAACAGATGG
*LaMVB34*-R	GCTTCTTGAGACAAGCGTGC
*LaNFYC*-F	ACATTGTTAATCGTTACAGCAAAGC
*LaNFYC*-R	TCCCATAGCCAGGTGATCCA
*LaDnaJA6*-F	GCAGTGGCTAAGCTAGCAGG
*LaDnaJA6*-R	TTTCCCAACCCAGTCGATCC
*LaDnaJ1*-F	GGAAATCCATTTGGTGGCGG
*LaDnaJ1*-R	CCAAGCTGACCTTGAGAGGG
*LaDnaJ20*-F	ATTACCATCATCATCAGGTGAGT
*LaDnaJ20*-R	GTTTTGACCCATCCTAGCAGC
*LaNAC21*-F	AGCCATGGGATATTCCTGAAACAG
*LaNAC21*-R	TCTCCCTTTTGGTGCTCTTCC
*LaNAC22*-F	CCCAAAGGAAATAAAACTGAGTGGG
*LaNAC22*-R	ACACAAAACCCAATCTTCCTTGG
*LaNAC35*-F	CCATGGGAATTGCCTGATTTGT
*LaNAC35*-R	AGGCCTTCCTCCACTACCAT

## Data Availability

The data were uploaded to the NCBI SRA database (SUB14514279).

## Supplementary Materials

The following supporting information can be downloaded at: https://www.mdpi.com/article/10.3390/plants14223408/s1, Figure S1: RT-PCR identification of the GsHZ4 overexpression strain; Table S1: The upregulated expression genes compared between Group A and Group B; Table S2: The down-regulated expression genes compared between Group A and Group B; Table S3: The upregulated expression genes compared between Group A and Group C; Table S4: The down-regulated expression genes compared between Group A and Group C; Table S5: The upregulated expression genes compared between Group A and Group D; Table S6: The down-regulated expression genes compared between Group A and Group D; Table S7: The upregulated expression genes compared between Group A and Group E; Table S8: The down-regulated expression genes compared between Group A and Group E; Table S9: The gene ID of the transcription factor.
