# Supplementary figures and images for: Leveraging Transcriptome Insights and GsHZ4 Gene Expression to Improve Alkaline Tolerance in Lupinus angustifolius
Source: Plants (Basel). 2025 Nov 7;14(22):3408. doi: 10.3390/plants14223408 (PMC12656323; doi:10.3390/plants14223408)

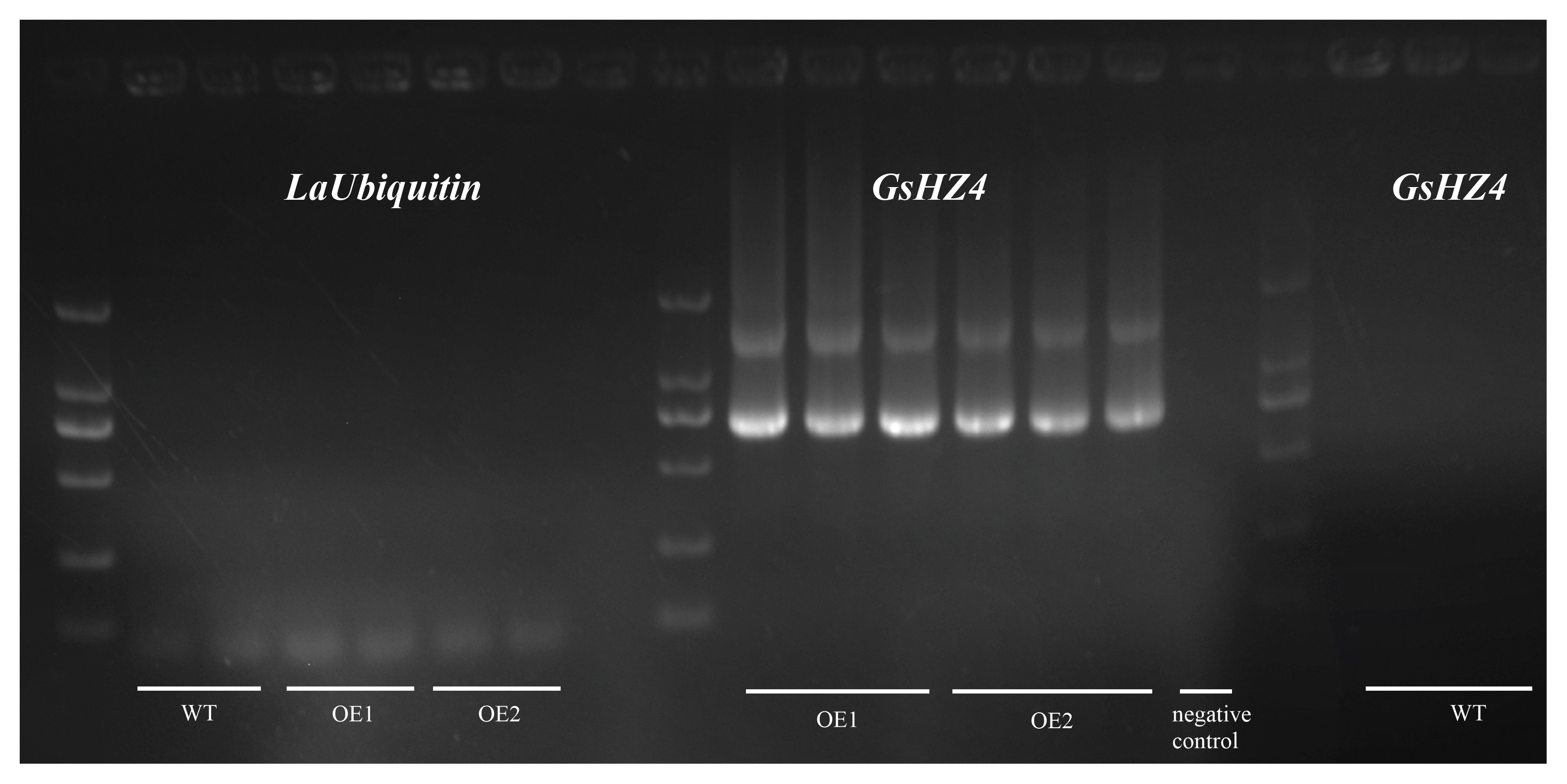

Supplement: Supplementary file 1 [file plants-14-03408-s001.zip › Figure S1.tif]
